# Changes in macrophage immunometabolism as a marker of skeletal muscle dysfunction across the lifespan

**DOI:** 10.18632/aging.204750

**Published:** 2023-05-25

**Authors:** Norika Liu, Joshua T. Butcher, Atsushi Nakano, Andrea del Campo

**Affiliations:** 1 Department of Cell Physiology, The Jikei University School of Medicine, Tokyo, Japan; 2 Department of Molecular Cell and Developmental Biology, University of California Los Angeles, Los Angeles, CA 90095, USA; 3 Department of Physiological Sciences, College of Veterinary Medicine, Oklahoma State University, Stillwater, OK 74078, USA; 4 David Geffen Department of Medicine, Division of Cardiology, University of California Los Angeles, Los Angeles, CA 90095, USA; 5 Laboratorio de Fisiología y Bioenergetica Celular, Facultad de Química y de Farmacia, Pontificia Universidad Católica de Chile, Santiago 7810000, Chile

**Keywords:** immunometabolism, macrophage, sarcopenia, aging, scRNA-seq

## Abstract

One of the most pronounced changes in the elderly is loss of strength and mobility due to the decline of skeletal muscle function, resulting in a multifactorial condition termed sarcopenia. Although significant clinical changes begin to manifest at advanced ages, recent studies have shown that changes at the cellular and molecular level precede the symptomatology of sarcopenia. By utilizing a single-cell transcriptomic atlas of mouse skeletal muscle across the lifespan, we identified a clear sign of immune senescence that presents during middle age. More importantly, the change in macrophage phenotype in middle age may explain the changes in extracellular matrix composition, especially collagen synthesis, that contributes to fibrosis and overall muscle weakness with advanced age. Our results show a novel paradigm whereby skeletal muscle dysfunction is driven by alterations in tissue-resident macrophages before the appearance of clinical symptoms in middle-aged mice, providing a new therapeutic approach via regulation of immunometabolism.

## INTRODUCTION

Society as a whole is experiencing increases in longevity. Indeed, the United Nations and World Health Organization estimates that the population greater than 60 years old will double in the next 30 years and reach a total of 2.1 billion people in 2050 [1]. Aging is directly associated with the time-dependent progressive loss of physiological integrity, increased frailty, and susceptibility to diseases [2]. Moreover, one of the most pronounced changes in the elderly is the loss of mobility and physical capacity due to skeletal muscle function decline, a term known as sarcopenia. This disability is independent of ethnicity, age, morbidity, obesity, income, or health behaviors and translates into a loss of independence in the elderly [3]. It begins approximately at 30 years of age and results in between 3-8% of muscle mass lost each year, with up to 40% of muscle mass loss after attaining 80 years of age [4]. Although quantifiable physical changes begin to manifest at advanced ages, recent studies have shown that changes at the cellular and molecular levels precede the symptomatology of sarcopenia [5–7]. As such, it would be advantageous to determine the early mechanistic underpinnings accompanying sarcopenia in the aged, with the prospect that maintenance of healthy skeletal muscle in the aged results in increasing healthspan along with the accompanying increase in lifespan.

The causes of sarcopenia have been investigated extensively and several hypotheses have been presented, with the usual suspects including; oxidative stress and inflammation, hormonal deficits, loss of neuromuscular junctions, mitochondrial dysfunction, and adiposity [8]. These studies have largely been inconclusive, making the mechanisms that cause sarcopenia still a matter of debate [9].

In the last decade, a new target has risen in prevalence, namely that immune cells may play crucial roles in an aging phenotype [10]. Historical evidence has highlighted that macrophage function in skeletal muscle is not confined to phagocytosis but also active participation in tissue repair and homeostasis [11]. Research has also highlighted that immunologic and metabolic pathways are intricately linked. Studies examining metabolic reprogramming of macrophages have proposed that the M1 phenotype rely mainly on glycolysis to obtain energy, while M2 macrophages obtain their energy by enhanced oxidative metabolism, triggering pro-inflammatory and anti-inflammatory properties respectively. Nevertheless, this classification is further nuanced as there may be metabolic differences between the same inflammatory program wherein M2 macrophages can be further classified into 4 distinct subtypes (M2a, M2b, M2c, M2d), meaning that modifications in macrophage intrinsic metabolism can alter their interaction with surrounding tissues [12]. Injury and subsequent repair of organ systems mediated by macrophages must have an appropriate balance between the M1/M2 phenotype for homeostasis to be restored.

Tissue-resident macrophage (TRM) populations are acquired early in development mostly from yolk sac or fetal liver erythromyeloid progenitor cells (EMPs) and canonically considered to be M2-like [13]. Embryonic-derived TRMs have self-renewal capacity and persist into adulthood. In some diseases, TRMs are involved in pathogenesis by being replaced by infiltrating monocyte-derived cells [14]. For instance, in some cancers TRMs may act as a cause of malignant transformation by recruiting or altering other immune cells [15]. Recent evidence has demonstrated that TRMs in skeletal muscle are distinctive from those in other tissues as they are more heterogeneous and are composed of functionally diverse subsets correlating to their origins [16]. In this regard, Reidy et al. demonstrated impaired muscle regrowth in aged compared with young mice following disuse, which was characterized by divergent muscle macrophage polarization patterns and muscle-specific macrophage abundance [17]. However, the direct impact of skeletal muscle-resident macrophages on aging, and if there’s a causal link to skeletal muscle function, has not been determined. Our study identifies key changes in the transcriptomic profile of tissue resident macrophages in skeletal muscle and further, reveals that these changes begin in middle age, suggesting that changes in immunometabolism across the lifespan may be an identifiable therapeutic target for prevention of sarcopenia in the aged.

## RESULTS

### Immune senescence is one of the most apparent signatures in skeletal muscle aging

**Figure 1:**
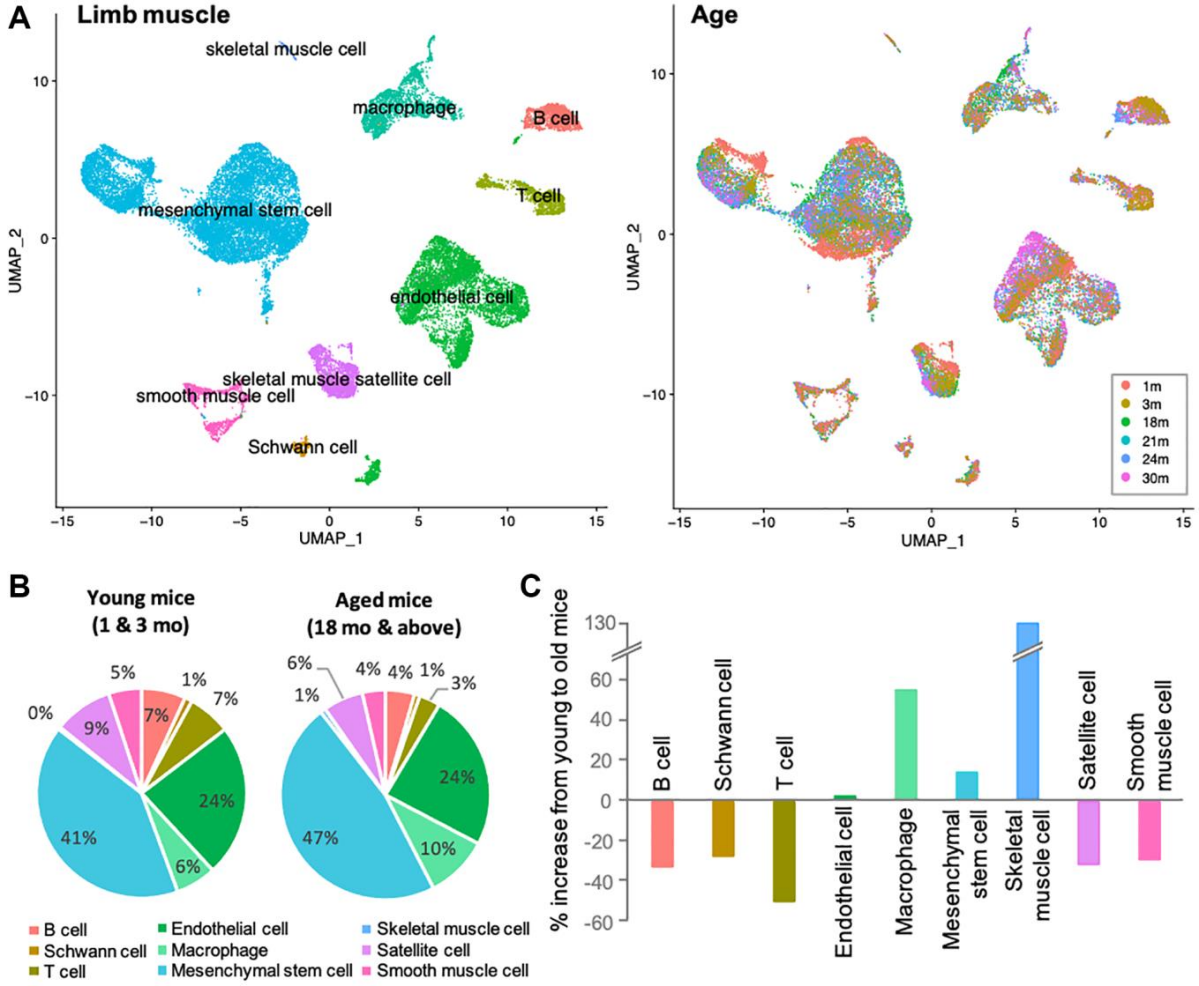
**Immune senescence is one of the most apparent signatures in skeletal muscle aging.** (**A**) UMAP representation of single-cell expression in limb muscles of mice of different age groups. Left UMAP indicates each cellular annotation. Right UMAP shows the cellular compositions of each age group. (**B**) Pie charts shows the percentage of various cells in the limb muscles of young mice (1 and 3-mo-old) and old mice (≥18-mo-old). Color coordination of cell types is consistent to left UMAP in (**A**). (**C**) Bar graph shows the % increase from 3-mo to 18-mo. B and T cells, Schwann cells, satellite cells and smooth muscle cells were reduced, while macrophages and mesenchymal stem cell increased. Note that the marked increase in skeletal muscle cells was due to a difference of 0% to 1%, and should be ignored from the data interpretation. Color coordination of cell types is consistent to left UMAP in (**A**).

Skeletal muscles are composed of various cell types, including myocytes, mesenchymal stem cells, endothelial cells, smooth muscle cells, muscle satellite cells, neuronal cells, and immune cells. To determine which cells in skeletal muscle are more susceptible to the aging process, we used Tabula Muris Senis, a single cell RNA sequence (scRNA-sequence) data from 1-, 3-, 18-, 21-, 24-, and 30-month-old mice (Figure 1A) [18]. The data contained all cell types that limb muscle tissue should have, as described above. However, the number of skeletal muscle cells was strikingly low in the dataset, which may be due to loss in the process of preparing single cells. Although the detailed protocol includes a process of digestion and filtration sufficient to yield mononuclear cells, skeletal muscle cells are multinucleated in nature and were probably lost in this process [18, 19]. Therefore, the data is not suitable for analysis of skeletal muscle cells, but contains a large and representative number of other cell types and would be powerful for comprehensive analysis. To identify age-related changes, we first compared the proportions of each cell type in young (1 and 3 mo. old) and aged (≥18 mo.) mice, as one simple indicator (Figure 1B). The definition of aged mice was derived from previously published work [20, 21]. Aside from skeletal muscle (again, the marked increase in skeletal muscle ratio is only due to the change from 0% to 1%), most cell proportional changes were within a 50% increase or decrease. In aged mice, the ratios of B cells, Schwann cells, T cells, satellite cells, and smooth muscle cells decreased, while the ratio of macrophages increased (Figure 1C). Ratios of endothelial cells and mesenchymal stem cells did not alter significantly. If skeletal muscle cells are excluded from data interpretation for the reasons indicated above, the most significant changes would be seen in immune cells, namely B cells, T cells, and macrophages. Acquired immune system cells, represented by B and T cells, were abundant in young mice, whereas innate immune system cells, represented by macrophages, were enriched with age. These alterations in immune cells are known as immune senescence, which is associated with muscle dysfunction such as sarcopenia [22].

Thus, by comparing the ratios of individual cell types between young and aged mice, we found that the most dramatic differences existed in the immune cells. These findings led us to hypothesize that even under healthy conditions, the skeletal muscles of aging mice have a pronounced immune senescence, promoting a decline in skeletal muscle function.

### Proinflammatory monocyte-derived macrophages play a central role in muscle aging

**Figure 2:**
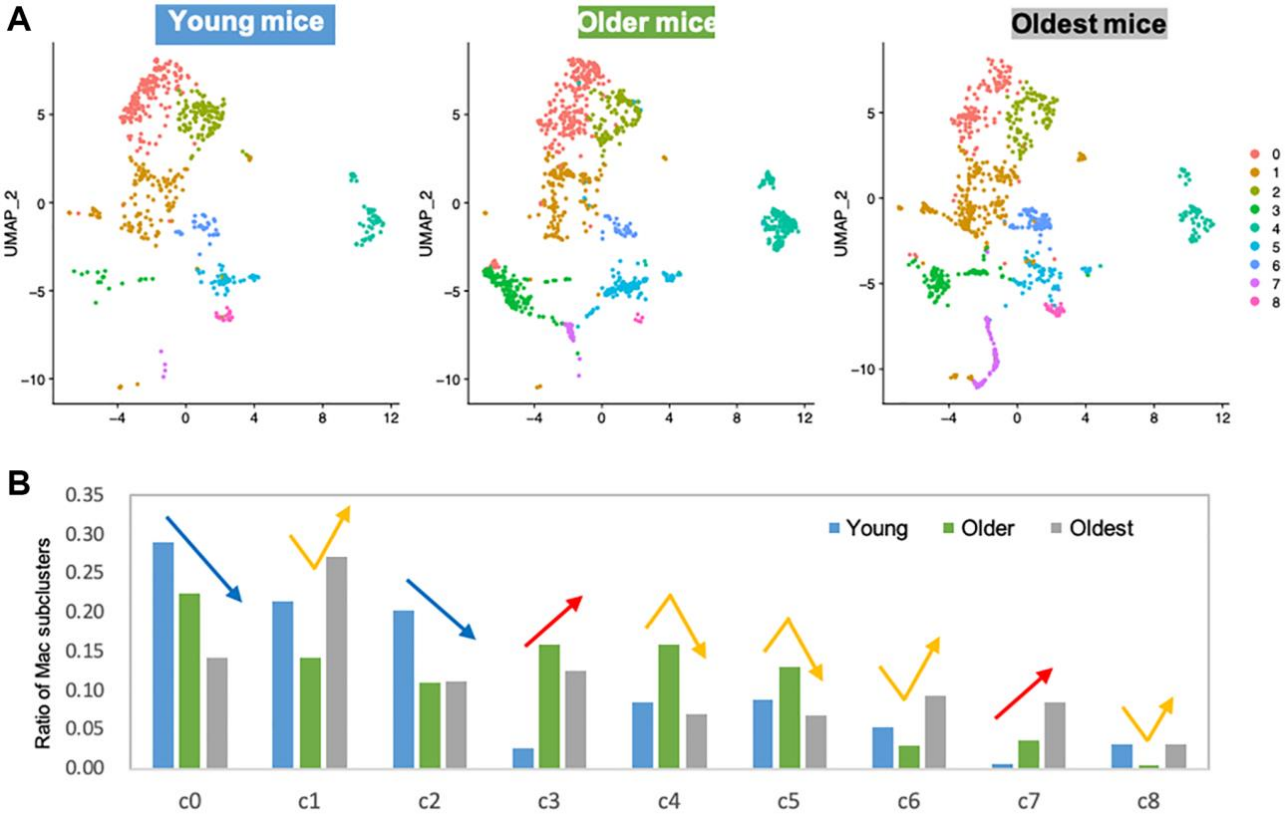
**Subclustering of macrophages shows clear trends of age-related decrease or increase.** (**A**) UMAP representation of macrophage subclusters in three age groups. Young mice: 1 and 3 mo. old, Older mice: 18 and 21 mo. old, Oldest mice: 24 and 30 mo. old. (**B**) Bar graph shows the ratio of individual subcluster macrophages present in each age group. Blue/red arrows are indicated on subclusters that had age-associated decrease/increase. Yellow arrows are indicated on subclusters that showed middle-age-specific tendency.

**Figure 3:**
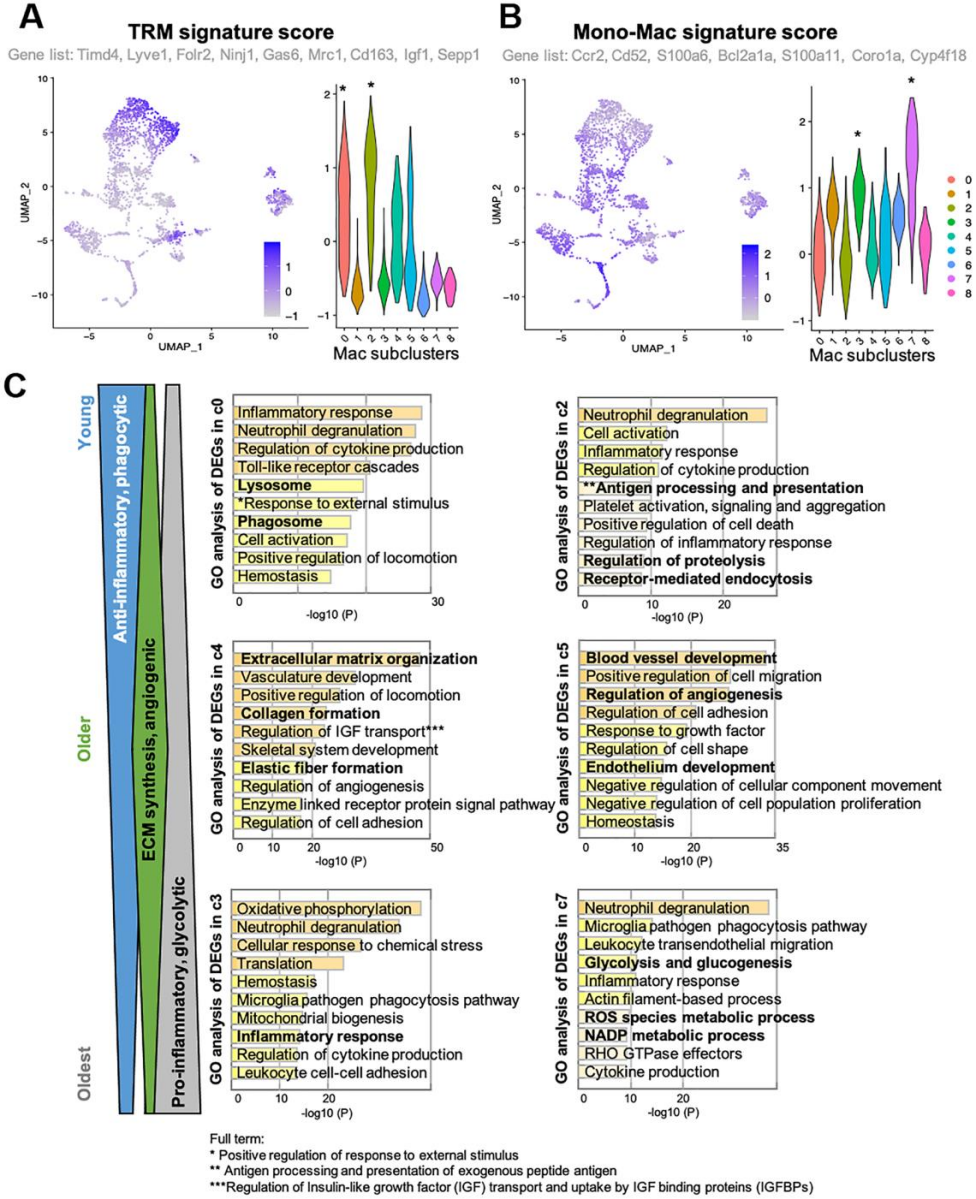
**Clusters with clear trends of age-related decrease or increase reflect their origin signatures.** (**A**, **B**) Feature plots and violin plots show signature scores of TRM or Monocyte-derived macrophage marker genes. Plots were generated using AddModuleScore function in R. Genes that were included as each signature score analysis are also indicated. Cell cluster of interest are indicated by ^*^in the violin plot. (**C**) GO analysis of DEGs in young mice-specific (c0 and c2), Older mice-specific (c4 and c5), and Old mice-specific (c3 and c7) subclusters. GO terms included in c0 and c2 suggests young mice-specific macrophage populations are more phagocytic, anti-inflammatory macrophages. GO terms included in c4 and c5 suggests older mice-specific macrophage populations are involved in ECM synthesis and angiogenesis. GO terms included in c3 and c7 suggests old mice-specific macrophage populations are glycolytic, pro-inflammatory macrophages.

In skeletal muscle, macrophages are known to play important roles in tissue homeostasis, repair, immunity, and pathophysiology [23, 24]. Alterations of macrophage profiles have been described in skeletal muscle of aged mice. Specifically, a study demonstrated that muscle regrowth in aged mice is impaired compared to young mice, and was characterized by macrophage polarization patterns and specific macrophage abundance [17]. Interaction between macrophages and muscle stem cell population was established by Wang et al. when they demonstrated that transplantation of young mice bone-marrow derived cells into aged mice prevented sarcopenia and age-related shifts in muscle fiber phenotype [10]. Thus, we hypothesized that the increase in certain macrophage population with aging is a contributor to functional decline in skeletal muscle. Macrophages phenotypes are highly diverse, with dynamic changes in response to the microenvironment. To examine the diverse phenotypic changes in macrophages with age, macrophage populations in the scRNA-seq data were sub-clustered and classified into nine clusters (Figure 2A and Supplementary Figure 1). Anti-inflammatory M2-type macrophage markers were enriched in subclusters 0 and 2 (Supplementary Figure 2A), whereas pro-inflammatory M1-type macrophage markers were evenly distributed (Supplementary Figure 2B). Age-wise representation of M2/M1-type macrophage signature score indicated that M2-macrophages tend to decrease as aging (Supplementary Figure 2C). However, M1-macrophage markers were consistent across the ages, suggesting that the typical classification of M1 and M2 types are not enough to interpret the data (Supplementary Figure 2D). Instead of classifying macrophage subtypes into M1 and M2 types, other approach for categorizing them is whether they are tissue resident macrophages (TRMs) or are monocyte-derived macrophages [16, 25]. This classification reflects their origins and functions. TRMs derived from erythromyeloid progenitors have a high capacity of self-renewal and so reside at the same location for a long time [25]. On the other hand, hematopoietic stem cells give rise to monocyte-derived macrophages. They are short-lived and are constantly replaced by circulating monocytes [25]. In some tissues, it has been observed that TRMs undergo apoptosis when inflammation occurs and monocyte-derived macrophages replace them [14]. A recent study revealed the core gene sets that represent either TRMs or monocyte-derived macrophages that are common across life time and in multiple organs including skeletal muscle [25]. In our analysis of tissue macrophages in skeletal muscles, when divided into young mice (1 and 3 mo. old), older mice (18 and 21 mo. old), and oldest mice (24 and 30 mo. old), there is a clear trend of age- related decrease or increase in numerous subclusters. Subclusters 0 and 2 decreased with age, while subclusters 3 and 7 increased with age (Figure 2B, blue and red arrows). These age-related decreasing/increasing trends were surprisingly concordant with TRM/monocyte-derived macrophage enrichment (Figure 3A, 3B). Further, gene ontology (GO) analysis was performed on differentially expressed genes (DEGs) of subclusters 0, 2, 4, 5, 3, and 7, which showed most remarkable decrease or increase with aging (Figure 3C). Macrophages in subclusters 0 and 2 (c0 and c2) were significantly enriched in the phagocytic phenotype, typical of an M2-like population (Figure 3C, upper two). Macrophages in subclusters 4 and 5 (c4 and c5) seemed to be involved in the extracellular matrix (ECM) formation and angiogenesis (Figure 3C, middle two). On the other hand, macrophages in subclusters 3 and 7 (c3 and c7), an oldest mice-specific population, were abundant in metabolic signatures such as glycolytic and oxidative stress-induced metabolic process (Figure 3C, lower two).

### Tissue macrophages in older mice may play an important role in ECM remodeling and may be involved in the onset of muscle aging

**Figure 4:**
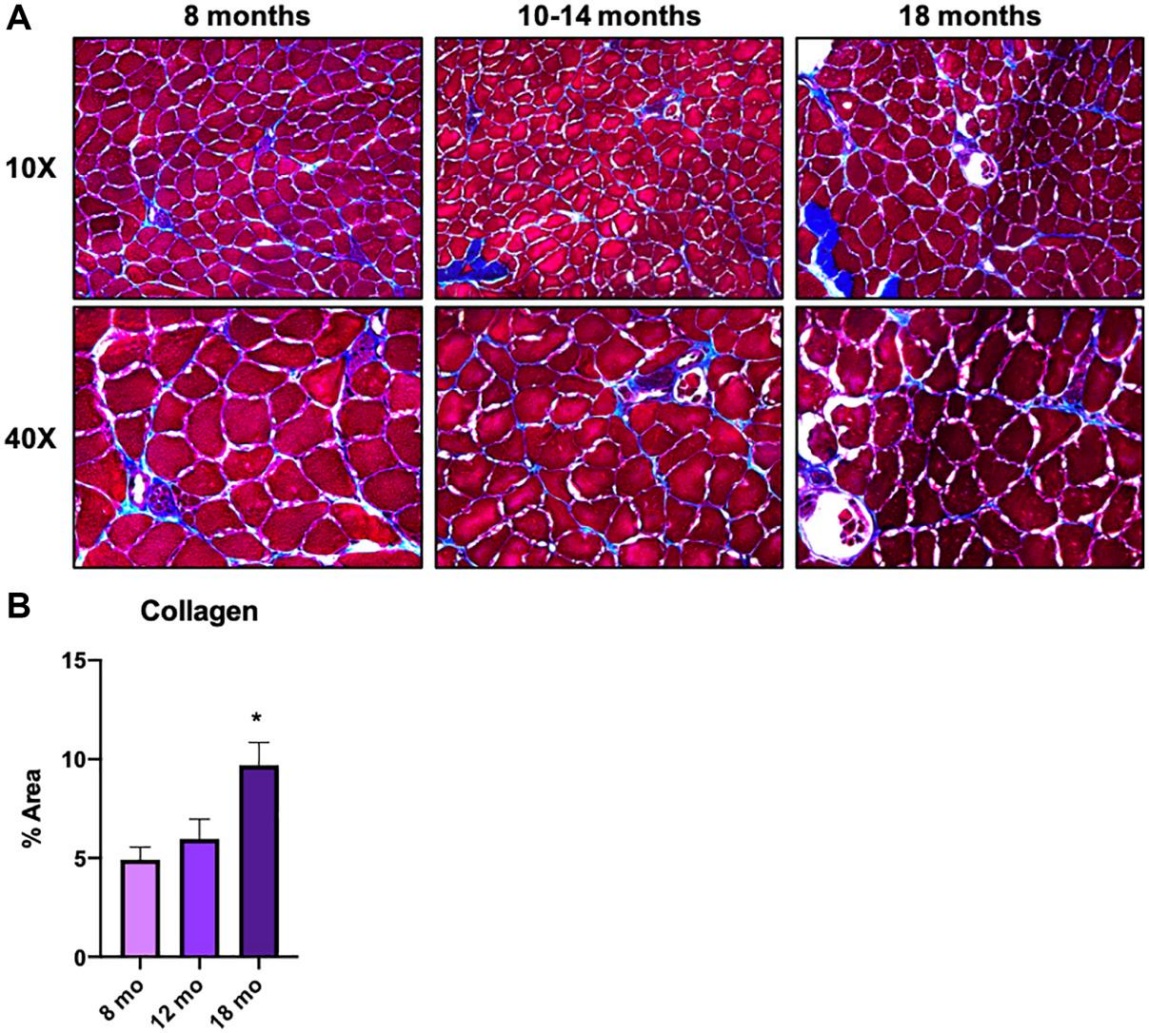
**Macrophages in middle-aged skeletal muscle tissue are involved in ECM remodeling and contribute to the onset of skeletal muscle aging.** (**A**) Gastrocnemius/soleus histologies with Masson Trichromic dye of 8 mo. old, 10-14 mo. old, and 18 mo. old mice. Both 10x and 40x magnification images are shown. (**B**) Bar graph shows the results of the quantification of collagen staining area in each age group. *N* = 3 Per group.

In the previous section, we examined macrophages that decrease or increase with age, while several macrophage subclusters showed specific proportional characteristics in older animals (18 and 21 mo. old), namely subclusters 1, 4, 5, 6, and 8 (Figure 2B, yellow arrows). From a clinically translatable perspective, changes in the tissues of older animals are important to enable early intervention. In fact, sarcopenia, a multifactorial syndrome of age-related loss of skeletal muscle mass and function, has been reported to begin in humans in approximately their 30 s [5]. Therefore, we again performed GO analysis on the differentially expressed genes in subclusters 4 and 5, which showed a pronounced increase only in older mice (18 and 21 mo. old), to understand their significance. Both subclusters included terms that related to vascular development and angiogenesis (Figure 3C, middle two). On the other hand, subclusters 1, 6, and 8, were strikingly enriched with ribosomal genes and overall were downregulated in the older groups (Supplementary Figure 3). The results for subcluster 4 are of particular interest due to the abundance of terms related to ECM formation. It is widely known that ECM of skeletal muscle is involved in mechanical functions, muscle repair and regeneration [26]. Excess or altered formation of ECM in aged muscles is known to contribute to tissue fibrosis, resulting in increased mechanical stiffness and decreased physical activity [27, 28]. Although studies suggest that collagen increases with age [27], others showed a decrease of collagen fiber tortuosity rather than collagen content or fiber orientation in the ECM of aged muscle [28, 29]. Further, the study reported that the age-related alteration of ECM drive muscle stem cell differentiation toward a fibrogenic lineage [28]. Our *in vivo* study using gastrocnemius/soleus muscles confirmed that collagen accumulation increases linearly with age and was more evident in 18-mo-old animals (Figure 4A and 4B). Taken together, the scRNA-seq data and *in vivo* analysis suggested a correlation between ECM remodeling, particularly collagen accumulation, and regulation by a macrophage subpopulation. As such, future research is warranted to determine if macrophage subpopulations can alter ECM remodeling and potentially serve as an early sign of muscle aging.

## DISCUSSION

The underlying mechanisms whereby aging is accompanied by sarcopenia continues to be an elusive target despite continued experimental interrogation, with increasingly concerning relevance as society faces a “global greying” phenomenon. Given the breadth of the cell populations within skeletal muscle itself, it is likely that the overall sarcopenic phenotype is driven by subtle and opposing alterations to multiple cell types throughout the organ itself. As such, sc-RNAseq analysis of whole muscle uniquely provides both a quantitative and transcriptomic cellular profile, providing a relevant systemic picture that can also provide insight into intracellular shifts that occur in an aging skeletal muscle. The present study focused on changes in the lifetime characteristics of tissue macrophages in skeletal muscle. Although a recent study has shown that skeletal muscle macrophages have age-associated subpopulations [30], what distinguishes our study from theirs is the diversity of ages covered and the fact that we found changes in macrophages in a completely unbiased manner, considering all cell types in skeletal muscle.

In the data used (GEO #GSE132042) we clustered a young male mouse population (1 and 3 months of age) for comparison of cell populations against an aged population (18–30 months of age). The middle age time point was especially relevant as, even though sarcopenia at this age is modest, the adaptations could provide innovative therapeutic targets, with the assumption that the underlying pathology has not manifested into irreversible functional outcomes [31–33]. While only rough estimates, the representative comparison between human populations would be 3–20 years (childhood-adolescence) and 56–80 (middle age-aged).

Our first observation (Figure 1C) is that that there is a substantial decrease in the cells that directly contribute to adaptive immunity, namely B cell and T cells. This supports evidence that aging does result in a reduction of lymphoid cell lineages, which are well characterized to play crucial roles in skeletal muscle regeneration and also accompanied by decreases in satellite cells, which are also observed [33]. Further, the loss of Schwann cells indicates that the neuromuscular junction (NMJ) is likely already undergoing degeneration and subsequent denervation [34]. Interestingly, smooth muscle cells also manifest a decrease with aging but are not accompanied by a similar decrease in endothelial cells. While speculative, this would suggest that the overall capillary rarefaction that occurs with aging and would directly impair perfusion/demand matching in skeletal muscle is driven by dysfunction to the upstream arteriolar tree or downstream venules [35, 36].

Importantly, the most substantial increase in a specific cell type is skeletal muscle macrophages, with aging resulting in almost a 60% increase in the overall population. To better evaluate the changes within the tissue-specific macrophage population across the lifespan, the macrophage data was subclustered (Figure 2A) into a young, older, and oldest groups. There were 9 identified subpopulations that emerged (Figure 2B). Remarkably, there are clear trends whereby the changes in the macrophage’s subpopulations are balanced by an opposing cluster (i.e., two downward trends (blue arrows) are balanced by two upward trends (red arrows). When further analyzed, these populations have very distinctive markers that define them as either tissue-resident macrophages (TRMs) or monocyte-derived macrophage markers. Subclusters that decrease with aging are characterized by TRM molecular signatures and subclusters that increase with aging are defined by monocyte-derived markers. Differential gene analysis (Figure 3C) revealed that, compared with young mice, older mice have a substantial shift in metabolic programming. We see a significant shift in the macrophage subpopulations in the young (M2-like, phagocytic, anti-inflammatory subpopulation) compared the oldest (M1-like, glycolytic, pro-inflammatory subtype). Age-related decrease in phagocytic macrophages has in fact been found to be one of the key features of immune senescence in many tissues [37–39]. First, based on the c0 and c2 subclusters, we see pretty substantial decreases in the transcriptome that regulates phagocytosis, and this begins with the older group. This is a key characteristic of the M2 phenotype, also known to play key roles in ECM component production and angiogenesis. Interestingly, while M2-like macrophages dominate in among in skeletal muscle and increase with aging, it appears that the phagocytic ability substantially decreases [40–42]. It is worth noting that, although we broadly define our clusters as M1 versus M2-like, that M2 macrophages are known to have metabolic flexibility and have both oxidative and glycolytic pathways intact. This concept perhaps explains the higher monocyte-derived signature in subcluster c3 accompanied by DEGs emphasizing oxidative phosphorylation. On the other hand, subcluster c7 has the highest monocyte-derived signature, along with DEGs that also show the glycolytic and inflammatory/ROS that accompanies M1-like macrophages, and clearly shows an upward trend with aging, a trend lacking from subcluster c3.

Finally, to examine potential interventional targets within the older group, we examined subclusters c4 and c5 as they both had characteristics that peaked during this time frame. There was significant upregulation of DEGs that emphasized both angiogenesis and extracellular matrix organization. This is conducive with other published data that suggests that TRM macrophages largely have an M2 phenotype. Further, it is well characterized that the oxidative M2 macrophage population (with an anabolic phenotype) supports and drives angiogenesis and tissue remodeling [43, 44]. However, regardless of the upregulation of the “regenerative macrophages” in the older group, there is still a significant deposition of collagen in the skeletal muscle itself, which we confirmed to be significant at 18 months of age (Figure 4B), and likely driving an overall fibrogenic phenotype in muscle with aging. Given that the subclusters 1, 6, and 8 are all dominated by ribosomal subunits, but are downregulated with the older group, this may suggest an inappropriate remodeling phenomenon is occurring, laying the proverbial groundwork for aging-related dysfunction later in life. Thus, these macrophages are either dysfunctional or undergo switching in the process of aging. Again, this directly supports previous work that demonstrates macrophage subpopulations drive overall collagen deposition and scarring, specifically via osteopontin, a glycoprotein with multiple functions regarding macrophage switching and known to be upregulated with aging [45, 46].

These alterations in intrinsic immunometabolism associated with aging are thought to affect systemic metabolism and, consequently, muscle function. In summary, the corresponding examination of age-related changes in macrophage subclusters revealed a clear decrease in TRM-like signatures (anabolic, anti-inflammatory) in combination with a clear increase in inflammatory (glycolytic, catabolic) signatures with aging.

Ultimately, this data supports a key concept, namely that dynamic changes in macrophage subpopulations may be key contributors to the overall progression of sarcopenia with aging. Further, these macrophage subpopulations are altered before the presentation of overt dysfunction, suggesting that regulation of subpopulations may be possible in terms of preventing or rescuing the progression of sarcopenia. Importantly, this can be extrapolated by examining current therapeutics that are frequently prescribed within the aging population (ex; resistance versus aerobic exercise, NSAID medication, metformin) to determine if some of the underlying positive impact is an effect on macrophage subtypes. Further, diseases that appear to indirectly alter skeletal muscle function (obesity, hormonal imbalance, viral infection, smoking) may be examined to see if the underlying driver of skeletal muscle dysfunction is acceleration of immune function toward an aging-like macrophage subpopulation [47].

Limitations of the study: the study has several limitations, the first being that the pathways identified by gene sequencing (which can overlap) need to be directionally validated, likely involving a challenging experimental paradigm incorporating immunometabolism, lineage tracing, and skeletal muscle function across the lifespan. Further, it should be noted that this experimental dataset is exclusive to the male sex. It is unlikely that females, especially considering reproductive capacity as a variable, would experience similar alterations to immunometabolism. Indeed, the definition of aging itself, along with the effectiveness of interventions, would likely be insufficient across sexes. Taken together, while significant questions remain, macrophage immunometabolism remains a promising target for both diagnostic and therapeutic interventions within skeletal muscle.

## MATERIALS AND METHODS

### scRNA-seq data analysis

scRNA-seq data are found in Gene Expression Omnibus (GEO) accession number GSE132042. Normalized rds file of limb muscles were downloaded from figshare (https://figshare.com/articles/dataset/tms_gene_data_rv1/12827615) and analyzed in R (v4.1). To convert the data into a format that can be analyzed by the Seurat package (v4.1), colData from sce (=single cell experiment) was entered as metadata. Data normalization was already done in the downloaded data. Cell annotations were applied according to the original data labeled “cell_ontology_class”. Cell counts of each cell type in each age group were used to calculate the percentage of that cell type presence. Uniform Manifold Approximation and Projection (UMAP) for a general non-linear dimension reduction was applied in all the figures because it is one of the most common visualization algorithms and it was also applied in the original study [18]. Signature genes of M1 and M2 macrophages were adapted from previous studies [48, 49], as well as TRMs and monocyte-derived macrophages [25]. The scores of selected signature gene expressions were projected on UMAP using AddModule function.

Macrophage clusters (clusters 4, 17, and 18) were subset and subclustered by recalculating resolutions at 0.3. Subclustered macrophages were classified into three age groups for further analysis: young (1 and 3 months), older (18 and 21 months), and oldest (24 and 30 months). Characteristics of the macrophage subclusters were compared using differentially expressed genes identified by the FindMarkers function. Marker genes for each subcluster were subjected to Gene Ontology (GO) analysis using Metascape (https://metascape.org/gp/index.html#/main/step1).

### Mice

Male C57BL/6J mice were housed in temperature-controlled rooms (21°C), on a 12-h light/dark cycle. All animal procedures conformed to the National Institutes of Health Guide for the Care and Use of Laboratory Animals and were approved by the Animal Research Committee at the Jikei University School of Medicine (approval no. 2020063) and the INTA, University of Chile and the Pontifical Catholic University.

### Histological processing and collagen staining

Serial cryosections (12 μm thick) from adult mice muscle were fixed using freshly prepared paraformaldehyde (4%) for 30 min and washed in distilled water. We performed a Masson trichromic technique to stain collagen. Histological preparations were examined by bright-field microscope (Leica microsystems DM 500 – camera ICC50W) and 5 images were obtained of each preparation. These ones were later analyzed with image J (NIH).

### Statistical analysis

Data of n mice (*n* = 3) were expressed as mean ± SE and analyzed by one-way ANOVA. *P* value < 0.05 was considered statistically significant (IC 95%). All statistical analyses were performed using GraphPad Prism 5.

## Supplementary Materials

Supplementary Figures
