# Supplementary material for: Changes in macrophage immunometabolism as a marker of skeletal muscle dysfunction across the lifespan
Source: Aging (Albany NY). 2023 May 25;15(10):4035–50. doi: 10.18632/aging.204750 (PMC10258037; doi:10.18632/aging.204750)
Supplement: Supplementary Figures [file aging-15-204750-s001.pdf]

## SUPPLEMENTARY FIGURES

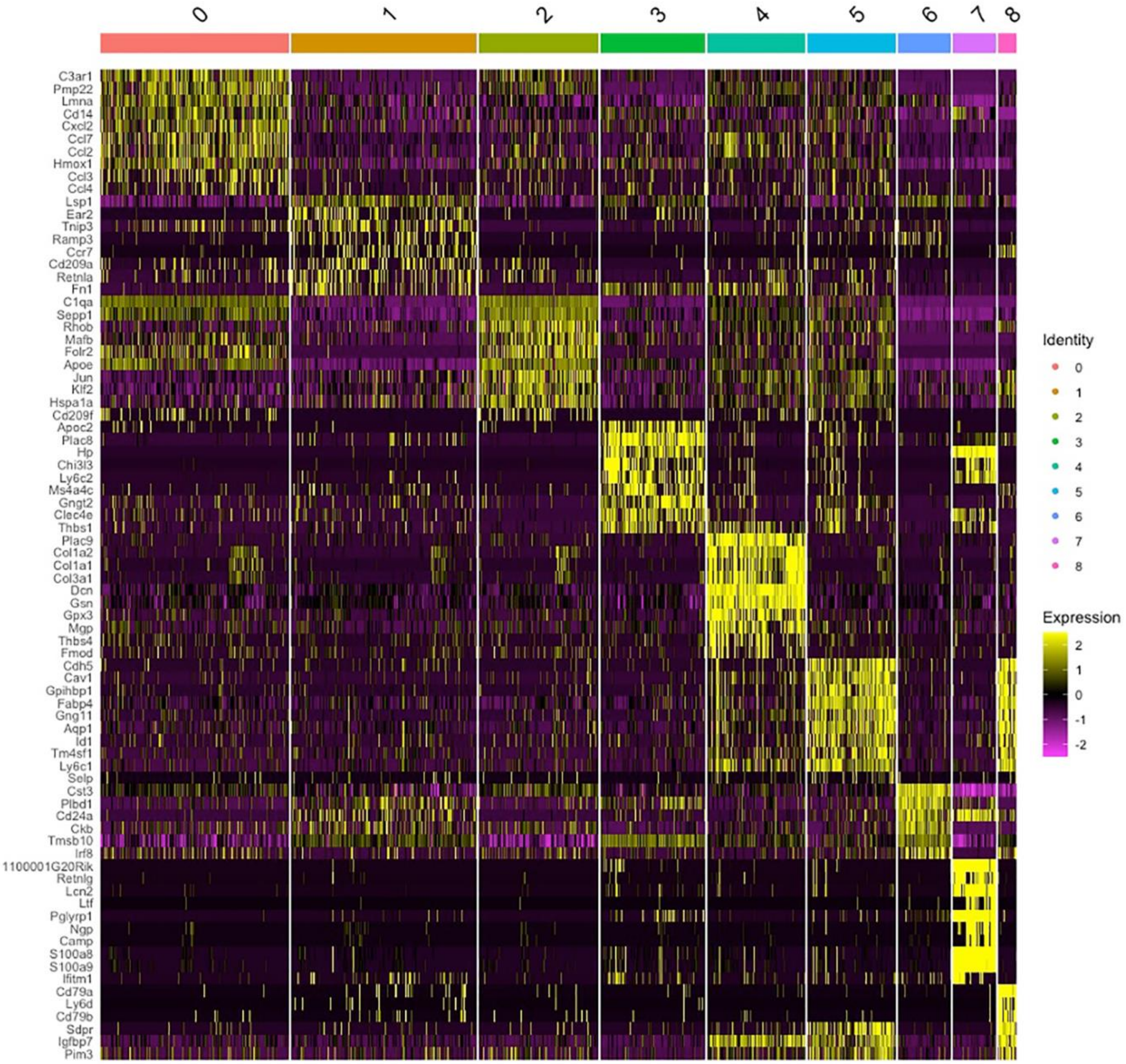


**Supplementary Figure 1. Heatmap of unsupervised clustering analysis featuring the top 10 discriminative genes per cluster.** Marker genes (defined by log2 fold change) of macrophage subcluster 0 to 8.

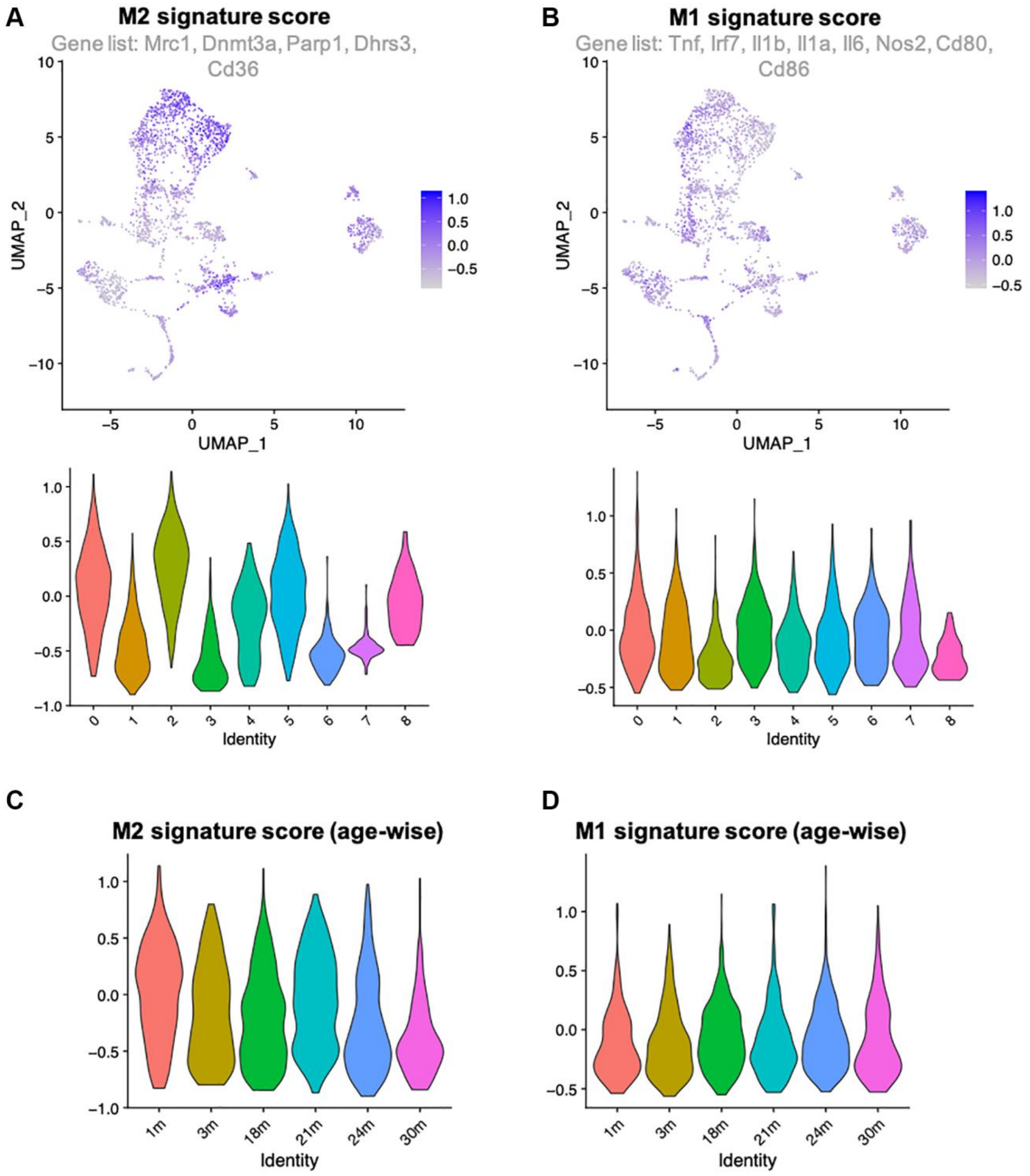


**Supplementary Figure 2. Signature scores of M1/M2 type macrophages.** (**A**, **B**) Feature plots and violin plots show signature scores of M2-type or M1-type macrophage marker genes. (**C**, **D**) Violin plots show age-wise signature scores of M2-type or M1-type macrophage marker genes. Plots were generated using AddModuleScore function in R. Genes that were included as each signature score analysis are also indicated.

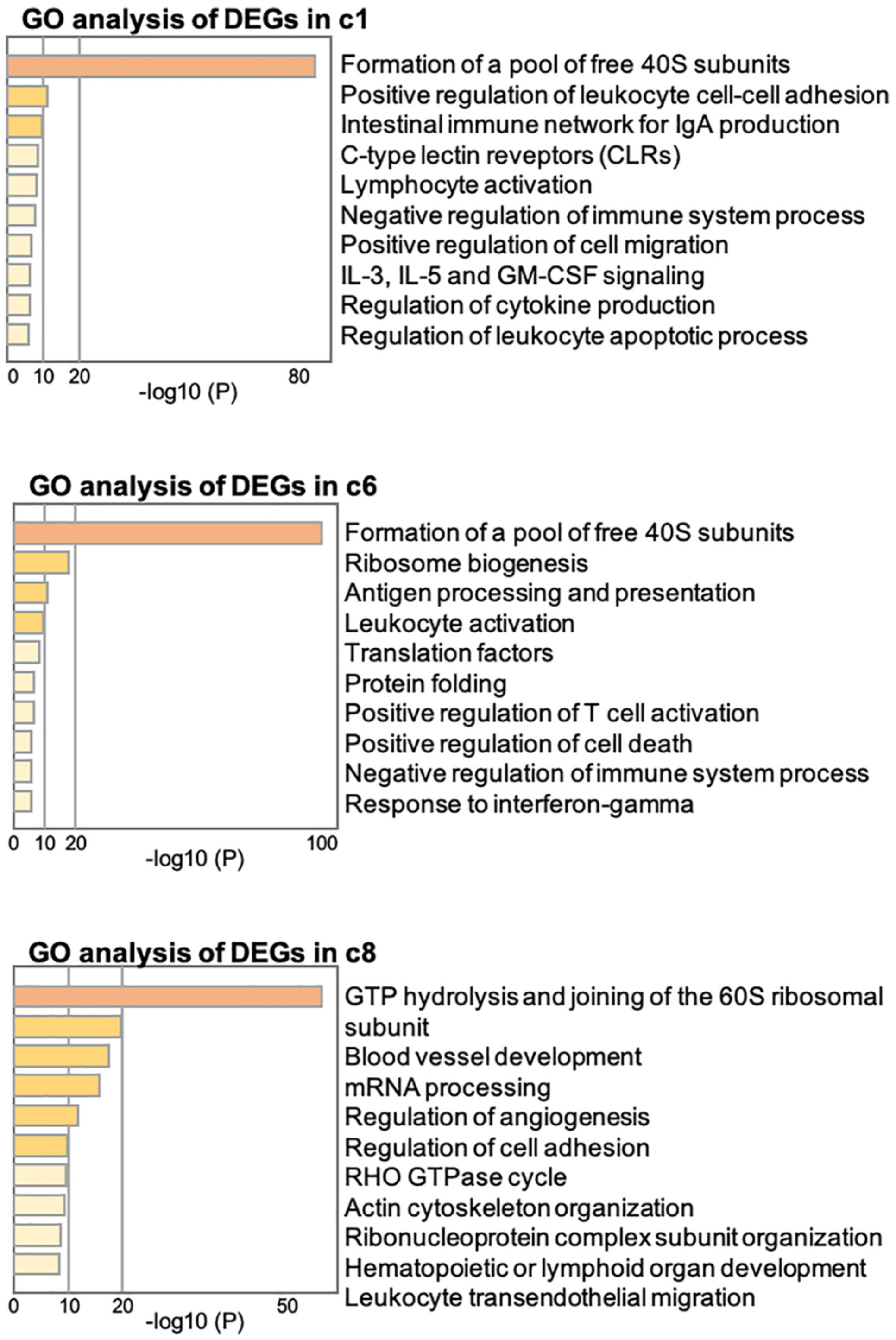


**Supplementary Figure 3. Genetic characteristics of macrophage subclusters specifically low in middle-aged skeletal muscle tissue.** GO analysis of DEGs in subclusters 1, 6, and 8. The top 10 most significant GO terms are listed and the X-axis is shown as Log *p*-value (−log10 (P)). Biological terms associated with ribosomal subunits were found to be strikingly increased in all three subclusters.
